# A Universal Cassette-Based System for the Dissolution of Solid Targets

**DOI:** 10.3390/molecules26206255

**Published:** 2021-10-16

**Authors:** Gabriele Sciacca, Petra Martini, Sara Cisternino, Liliana Mou, Jonathan Amico, Juan Esposito, Giancarlo Gorgoni, Emiliano Cazzola

**Affiliations:** 1 Legnaro National Laboratories, National Institute for Nuclear Physics, 35020 Legnaro, Italy; sara.cisternino@lnl.infn.it (S.C.); liliana.mou@lnl.infn.it (L.M.); juan.esposito@lnl.infn.it (J.E.); 2 Department of Industrial Engineering, University of Padova, 35131 Padova, Italy; 3 Department of Translational Medicine, University of Ferrara, 44121 Ferrara, Italy; mrtptr1@unife.it; 4 Cyclotron & Radiopharmacy Department, Sacro Cuore Hospital, 37024 Negrar, Italy; jonathan.amico@sacrocuore.it (J.A.); giancarlo.gorgoni@sacrocuore.it (G.G.); emiliano.cazzola@sacrocuore.it (E.C.)

**Keywords:** dissolution system, radiopharmaceutical, solid targets, automation, radiometals production, cyclotron production, radiochemistry, manganese-52, zirconium-89, technetium-99m

## Abstract

Cyclotron-based radionuclides production by using solid targets has become important in the last years due to the growing demand of radiometals, e.g., ^68^Ga, ^89^Zr, ^43/47^Sc, and ^52/54^Mn. This shifted the focus on solid target management, where the first fundamental step of the radiochemical processing is the target dissolution. Currently, this step is generally performed with commercial or home-made modules separated from the following purification/radiolabelling modules. The aim of this work is the realization of a flexible solid target dissolution system to be easily installed on commercial cassette-based synthesis modules. This would offer a complete target processing and radiopharmaceutical synthesis performable in a single module continuously. The presented solid target dissolution system concept relies on an open-bottomed vial positioned upon a target coin. In particular, the idea is to use the movement mechanism of a syringe pump to position the vial up and down on the target, and to exploit the heater/cooler reactor of the module as a target holder. All the steps can be remotely controlled and are incorporated in the cassette manifold together with the purification and radiolabelling steps. The performance of the device was tested by processing three different irradiated targets under different dissolution conditions.

## 1. Introduction

Radioisotopes (RIs), largely used worldwide in diagnostic imaging procedures in the fields of oncology, neurology and cardiology, are currently produced by medical cyclotron accelerators, starting from the irradiation of a specific target [1]. The growing number of cyclotrons of different energies installed worldwide has given a strong impulse to the production of conventional and emerging radionuclides for medical applications [2,3,4,5,6,7,8,9,10,11,12,13,14]. In particular, the great advantage of using medical cyclotrons is the possibility to produce the medical radionuclide of interest on site and on demand. Recently, the technological advancement in the radionuclides cyclotron-based production sector has encouraged the use of novel radioisotopes (mainly radiometals) in medical applications, for implementing the so-called personalized medicine approach. In particular, the strength of this approach relies on the possibility of selecting patients responding positively to the targeted treatment by performing a preliminary diagnostic imaging using the same radiopharmaceutical (theranostic approach) [15,16,17,18].

The most used RIs for PET (Positron Emission Tomography), such as ^18^F and ^11^C, are produced by liquid or gas targets, whereas the availability of radiometals through a cyclotron-based production requires the use of solid targets. This kind of target is generally composed by a pellet of the desired material, usually costly isotopically enriched material either in metal or oxide form, bonded to a backing plate or encapsulated in a holding shell [19,20,21]. Liquid target is also an alternative for radiometal production, certainly preferred in case of short half-life radionuclides, necessitating fast irradiation and processing chemistry [22]. However, limitations in the target concentration dissolved in the acidic solution lead to low production yield. In addition, this process is not exempt from issues related to the corrosiveness of the acidic target solution and possible gas formation [23,24,25]. On the other hand, despite the higher production yield achievable in choosing a solid target-based production, it requires important technological and structural investments for target manufacturing, specific target station, complex automated delivery systems and a dedicated post-irradiation target dissolution system [15,26]. This is in addition to the need to recover the costly enriched material.

Research and development on solid target technologies as well as commercial interest is very dynamic and is evolving very rapidly. Nevertheless, regarding target dissolution systems, to our knowledge mainly three devices are available to date on the market for solid target treatment (IBA-Pinctada^®^ metal [27], ARTMS_ QIS^®^ [28], and Comecer-ALCEO [29]). These are all self-standing systems independent from the synthesis module. Each system is suited to the characteristics of the target provided by the same company and not adaptable to alternative solutions. In some cases (e.g., ALCEO retrofit [29]) the target can be processed directly at the irradiation site to avoid the transfer of the solid target to the radiochemistry lab. From this point of view, other research prototypes have been reported in the literature. Gelbart and Johnson have recently proposed a solid target system with in situ target dissolution featuring heating up to 100 °C, and gas bubbling agitation [30]. Similarly, Beaudoin et al. also developed an in-vault system solid target dissolution system [31].

Having the opportunity to work with a medical cyclotron already equipped with a solid target station and a pneumatic transfer system to the radiochemistry lab, the purpose of this work was the development of a simple and efficient solid target dissolution system compatible with commercial cassette-based synthesis modules. In this way it would be possible to perform the radiochemical processing, from the dissolution to the labelling, all at once using a single remotely controlled device. Keeping the system compact allows containing all the process in a single hot cell, lowering the probability of external and operator contamination. At the same time, this reduces the processing time and maximizes the recovery yield thanks to the absence of wasteful transfers from one system to another. The entire process, starting from dissolution up to radiopharmaceutical formulation, can be applied continuously.

In this regard, a specific solid target dissolution system has been developed in a collaboration between the LARAMED (LAboratory of RAdionuclides for MEDicine) group of the Italian National Institute for Nuclear Physics (INFN), at the Legnaro National Laboratories, and the Sacro Cuore Don Calabria Hospital (SCDCH), located at Negrar di Valpolicella, VR, Italy [32]. The idea of this new reactor originates from the INFN WO/2019/053570 patent [33], describing the technology for manufacturing solid targets by Magnetron Sputtering technique. Indeed, this patent also includes a dissolution reactor system based on open-bottomed vial, which was originally applied on a semi-automatic prototype system used for ^99m^Tc and ^64^Cu dissolution and recovery [8,34].

In this paper we describe the developed system and tests performed with three different irradiated targets to demonstrate the performance of the system under different dissolution conditions.

## 2. Materials and Methods

### 2.1. Irradiated Targets

**Figure 1:**
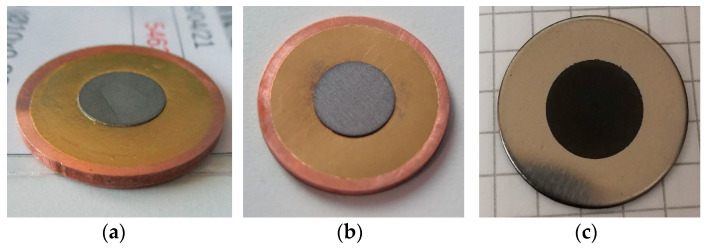
Mo (**a**), Cr (**b**), and Y (**c**) targets prepared with SPS technique, prior to irradiation.

Yttrium, chromium, and molybdenum metal targets have been prepared by Spark Plasma Sintering (SPS) [21]. This technique allows the sintering of a pellet starting from powder, and bonding it to a different material without the need of a filler [35,36]. In this work, Y targets were manufactured in one step: ^Nat^Y disc (Ø 12 mm, thickness 150 µm, purity 99%, Goodfellow) was bonded to a Nb backing disc (Ø 23.5 mm, thickness 1.7 mm, purity 99.99%, Goodfellow). Chromium and Molybdenum targets were prepared in 3 steps: First, green pellets of Cr or Mo (Ø 10 mm, thickness 400 µm and 280 µm, respectively) were prepared starting from the powder form; then, an inert Au foil (Ø 20 mm, thickness 25 µm, purity 99.95%, Goodfellow) was bonded to an Oxygen-Free High thermal Conductivity (OFHC) Cu backing disc (Ø 23.5 mm, thickness 1.7 mm, purity 99.95%) by SPS; finally, Mo or Cr pellets were press-bonded to the backing plate system (Au/Cu) by SPS. SPS machine prototype at University of Pavia (Italy) and a commercial machine (Dr. SINTER^®^ SPS1050, Sumitomo Coal & Mining Co. Ltd., now SPS Syntex Inc., Tokyo, Japan) were used. Manufactured targets are shown in Figure 1.

The target sizes fit the target station of ACSI TR-19 Cyclotron, whose details can be found in [37] and is installed at SCDCH where the studies were performed.

### 2.2. Design of the Reactor Components

The reactor was devised so as to be compatible with the electro-mechanics of commercial synthesis modules. The developed system fundamentally consists of a series of adaptors able to exploit the original purpose of some components of commercial modules.

The concept was tested on the Eckert & Ziegler (E&Z) Modular Lab and Trasis AllinOne commercial modules. These devices are based on disposable cassettes and have a suitable number of valves and components to implement the dissolution process together with the following purification and synthesis protocol. In particular, stepped motors and heated reactors are present. In the original modules’ configuration, they are intended for the movement of syringe drivers/activity plunger and the kinetics regulation of the chemical process, respectively. In both modules these components are conveniently located to implement a dissolution process of solid targets: in the proposed configuration, the stepped motor is used for the movement of a dissolution vial by means of a vial-holder and mounting rods, while the reactor’s heater is used to plug a target holder suitable for coin-shaped targets.

Hence, the design of the components was performed with the Computer Aided Design tool Solidworks^®^, after the geometry of the available modules had been reconstructed with the same software. All the parts were made of materials easily washable and as much as possible inert to the strong dissolution condition required for the radiometal isotope recovery. Since the system components are principally made of metallic materials, they were manufactured with Computerized Numerical Control machines. The pieces realized for this purpose are listed in Table 1, and a description of the various parts is hereafter reported.

#### 2.2.1. Reactor Dissolution Vial

This component, where the dissolution reaction process occurs, is most of the time in contact with strong acid solvents. For that reason, it must be made of inert material. In particular, three different materials were chosen for the proof of concept:

- Borosilicate glass;
- Quartz;
- PEEK.

**Figure 2:**
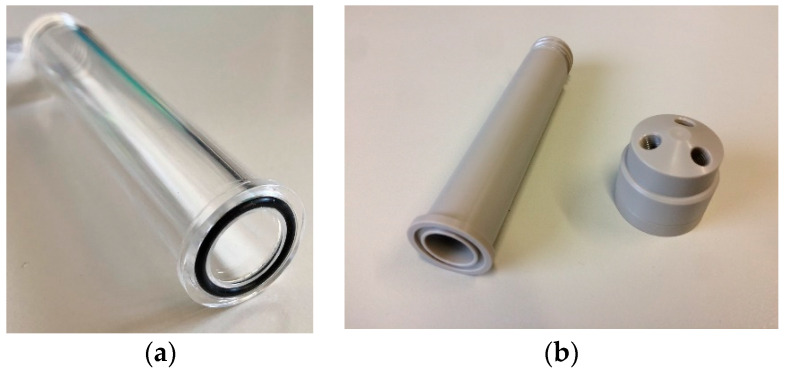
(**a**) Reaction vial made of borosilicate glass with mounted O-ring; (**b**) PEEK vial, with the PEEK Vial Head.

Indeed, these materials offer different inertness to acidic solutions. While PEEK vials could be machined in house, the ones in glass and quartz were manufactured by the French company Intellion S.a.r.l (Paris). The top of the vial was shaped to screw with the Vial Head for 3 connectors 1/4″, shown in Figure 2b, provided as accessories of E & Z modules. This allows the reaction vial to be connected with the cassette by standard tubing and connectors used in these kinds of applications. By contrast, the vial bottom part has a cavity dedicated to installation of a 15.08 mm ID (Internal Diameter) × 2.62 mm thick O-ring, used to seal the reactor with the backing disc. In this way, only the target material will be dissolved, thanks to the inertness of the backing plate.

#### 2.2.2. Vial-Holder and Mounting Rods

These components, made either in machined aluminium alloy 6082 or stainless steel in relation to the desired rigidity, were needed to connect the reactor vial to the stepping motor in order to perform the vial movement. Indeed, the vial could be kept suspended without slipping down owing to the contact of its holder with the cap, while it also allowed the vial to be held in contact with the target when pushed against it. Therefore, the component is structured in such manner as to facilitate the vial insertion and, in case of a transparent vessel, to allow some visibility inside it during the chemical attack.

**Figure 3:**
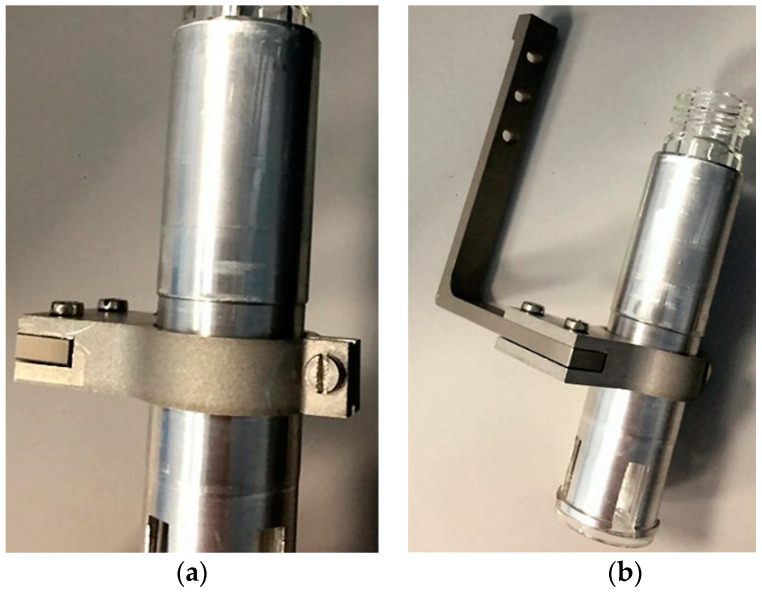
(**a**) Assembly of the vial holder; (**b**) mounting rods used for the E&Z module.

Since the moving elements of the modules are not well aligned with the target holder platform, connecting elements have to be adopted. Thus, an assembly of two rods was realized to mount the vessel holder to the syringe actuator. The images in Figure 3 illustrate the parts manufactured for the E&Z module. Slots were applied in correspondence to the bolted joints for the correction of possible misalignments. Enough thickness was then provided in the design of these elements to avoid their bending during the reactor sealing, which can cause solvent losses during operation. At the same time, their dimensions were adjusted to not cause collision with the modules’ components.

#### 2.2.3. Target Holder

**Figure 4:**
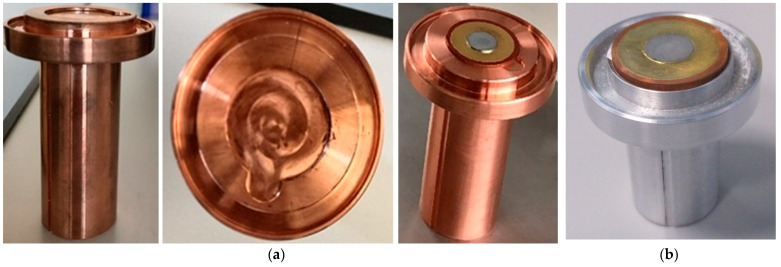
(**a**) Target holder made for the E&Z module shown in lateral view (left), upside view (middle), and with a sample target inserted (right). (**b**) Target holder made for the Trasis AllinOne, with the sample target inserted.

A dedicated target holder was developed in order to keep in position the target coin during the dissolution process. This component should have a good thermal conductivity, to efficiently heat the target when control on the reaction kinetics is desired. Its shape was devised as an extension of the reactor’s heater to the outside, while a dedicated cavity on the holder’s top allows the accommodation of the target. Moreover, as show in Figure 4, a groove surrounding the target slot is intended to confine possible losses from the vessel.

### 2.3. Dissolution Tests

Three different dissolution procedures were performed with the two reactors, installed on E&Z and Trasis systems, on three different irradiated targets (Y, Cr, and Mo) for the production of ^89^Zr, ^52^Mn, and ^99m^Tc, respectively.

Prior to each test, the sealing capability of the system was assessed to prevent possible losses of reactants during the dissolution. After passing the leakage test, the two synthesizers were then used for dissolution experiments of irradiated target at the conditions reported in Table 2. All chemicals and reagents involved in the purification process were of analytical grade, unless otherwise specified. Hydrogen peroxide 30% *w/w* and HCl 37% ACS reagents were purchased from Merck (Darmstadt, Germany).

All the dissolution steps were remotely controlled by the module’s dedicated software. To evaluate the dissolution efficiency, the target coins were always weighed before and after dissolution. Once dissolution was completed, visual inspection, gamma spectrometry, and activity measurement of the target solutions were performed for each production run. Gamma spectrometric measurements were performed using a High-Purity Germanium (HPGe) detector (Sw GENIE II Canberra, Meriden, CT, USA). The efficiency calibration was carried out in the energy window 17 keV to 1923.1 keV for three different geometries (Eppendorf 1ml; vial 5ml; vial 1ml) by using a multi-peak certified liquid source (containing the reference radionuclides ^241^Am, ^109^Cd, ^139^Ce, ^57^Co, ^60^Co, ^137^Cs, ^113^Sn, ^85^Sr, ^88^Y, and ^51^Cr), with Genie 2000 software. Activity measurement was carried out with a Capintec, Inc. dose calibrator, model CRC-25PET, periodically subjected to a quality control program. An appropriate factor was set for each isotope measured.

## 3. Results

### 3.1. Dissolution Reactor Assembly

The two dissolution reactors have been assembled, respectively, on the E&Z Modular-Lab and the Trasis’ AllinOne modules in a hot cell. All the parts were installed without particular difficulties on the commercial modules, and the slots available on the mounting rods allowed to align the vial with the target holder’s baseplate with the required precision. Connection of the reactor with the cassette is made easy thanks to the compatibility of the vial with the PEEK head.

**Figure 5:**
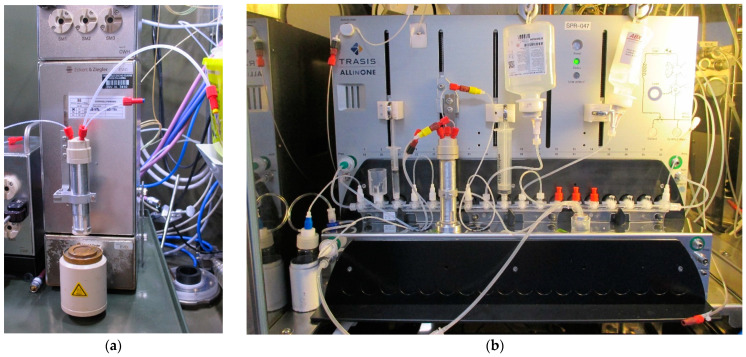
Pictures of the solid target dissolution system mounted on: (**a**) an E&Z module; (**b**) a TRASIS AllinOne module.

Figure 5 shows the developed systems implemented on the tested commercial modules, hosted inside the hot cell. In the dissolution process, after target placement, the reactor vial can be positioned on top of the coin target by controlling the movement of the syringe actuator. The pressure applied by the stepper motor adequately seals, by means of the O-ring, the vial during the chemical attack. Once the dissolution is completed, the solution can be pumped through the outlet channel connected to the top of the vial to the subsequent radiochemistry steps. All the operations can be remotely controlled with the respective modules’ software, and the system allows for the incorporation of the purification and radiolabelling steps within a single cassette manifold. Furthermore, the proposed reactor offers an intrinsic flexibility in processing targets having different thicknesses of the deposited target pellet.

### 3.2. Operational Testing

All the devices successfully passed the preliminary leakage tests without losses, thus proving to have enough rigidity to prevent possible release of radioactive liquid.

Following the automated transportation of the irradiated target from the cyclotron target station to the hot cell docking station, the target is transferred to the dissolution unit of the automatic system with tongs or telemanipulators. The target can be easily accommodated on the reactor heater baseplate with the target material pointing upwards in alignment with the bottom of the vial. The sealing of the vial on the target by the O-ring, during the chemical attack, allows for the selective dissolution of the target material minimizing the contact of the solution with the backing and avoiding liquid leakage. Liquid leakage never occurred during our dissolution tests. The dissolution of the irradiated target can be activated or hastened by heating the conductive baseplate. The use of a transparent glass vial allowed for the monitoring of the process, as was useful in the case of non-well-established procedures, like with the dissolution of Cr and Mo, whereas with Y target it was possible to use PEEK vial since the procedure was well-known.

Average weights of the target/backing ensembles and of the backing after target material dissolution are listed in Table 3. The amount of dissolved weight corresponds to the amount of the original target material. Therefore, in all three cases, all the target material was efficiently dissolved and removed from the backing in a reproducible manner.

Table 4 reports the measured activity at the end of dissolution (EOD) and rescaled to end of bombardment (EOB). These measurements can be compared with the theoretical predicted activity calculated at EOB by using the on-line tool ISOTOPIA [38]. The reported values are in agreement with the predicted activity and the gamma-spectrometry analysis. Furthermore, as additional confirmation of complete target dissolution, an additional chemical attack was performed with fresh solvents after dissolved target removal from the reactor in order to detect any residual activity remaining undissolved on the backing. From the following activity measurement, no relevant activity was found in any of the three solutions.

**Figure 6:**
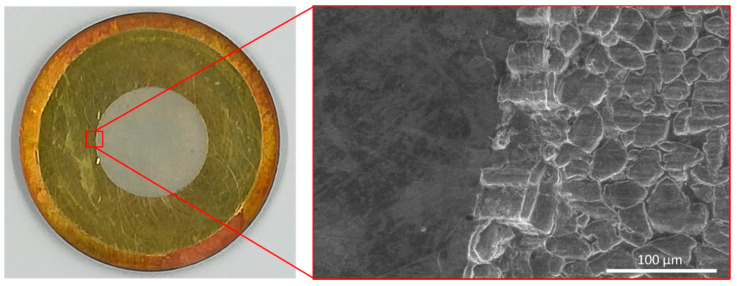
Left: image of the Au/Cu backing after the dissolution of Cr pellet; Right: SEM image of the Au layer surface at the boundary of the area where Cr was bonded.

As an example, Figure 6 shows a picture of Au/Cu backing after dissolution of not irradiated ^nat^Cr target analysed by SEM-EDS. The grooves on the right side of the SEM image correspond to the part where Cr pellet was attached. It is clearly visible that the Cr pellet was completely dissolved. Indeed, the EDS analysis detected traces of Cr (about 30% at.) that could be due to the bonding of Cr pellet to the Au layer. Considering that the SEM-EDS electron beam penetration depth is about 2 µm, the 30% at. of Cr corresponds to about 0.24 mg, approximately 0.1% of the Cr pellet mass, over the 1 cm diameter Cr spot, supporting the data shown in Table 3.

**Figure 7:**
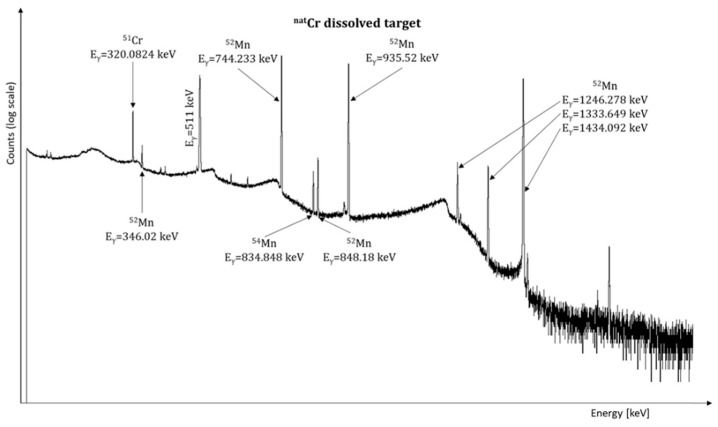
γ-spectrum of the dissolved ^nat^Cr target.

Below we report a spectrum of the ^nat^Cr dissolved target (Figure 7). In the spectrum only energy peaks corresponding to manganese and chromium isotopes are identifiable. In all the three performed dissolution studies, no radioactive contaminants coming from the backing material were detected. Minimum detectable activity (MDA) of the most prominent gamma lines for the contaminants ^197m/g^Hg and ^93m^Mo (potentially coming from the activation of Au and Nb backing materials, respectively) calculated from the spectra of the dissolved target solution for ^52^Mn, ^99m^Tc, and ^89^Zr are reported in Table 5.

## 4. Discussion

In this work the realization of a flexible solid target dissolution system to be easily installed on commercial cassette-based synthesis modules is described. The developed system allows for the dissolution of targets characterized by various diameters/thicknesses of the target material attached to the backing, regardless of their manufacturing protocol. The target material can be selectively dissolved and radiochemically processed in order to achieve an injectable radiopharmaceutical product of high purity. Both Cr and Mo targets were processed with the E&Z module by using transparent borosilicate glass vials enabling the visualization inside the vessel during the chemical attack to monitor the reaction. At the same time, dissolution of Y irradiated target was performed with a Trasis module using a PEEK vial since the dissolution procedure was standard and already established. Both vials correctly fitted with the cap and reactor ensuring a proper system operation. This demonstrates the possibility of manufacturing this particular design of open-bottomed vial with different materials (from PEEK to quartz). Moreover, the system properly works also changing the components’ material, both vial or reactor block, which can be selected according to the needs of thermal conductivity, resistance, and chemical inertness to the solvents involved in the process.

The dissolution system configuration has been tested on both commercial modules considered in this study and was successfully used to perform the dissolution of chromium, molybdenum and yttrium targets used for the ^52^Mn, ^99m^Tc, and ^89^Zr production, respectively. The system is versatile, since it can be used with targets made with different manufacturing techniques and may be adapted with different cassette-based commercial automatic modules (e.g., Eckert & Ziegler Modular-Lab and the Trasis’ AllinOne). Thanks to this system, the dissolution of the target can be remotely controlled, directly connected, and totally integrated with the separation and purification processes, keeping the operations safe and clean.

The use of a unique automated system for dissolution, separation, purification, and labelling will sharply decrease the radiation exposure of operators in handling high radioactive materials and, secondly, will definitely contribute to making the whole process much more reproducible, faster, and traceable, minimizing environmental contamination. That is indeed a key prerequisite for attaining a clinical-grade quality for the recovered radioisotope and radiopharmaceutical.

It is thus possible, for those who already own synthesis modules like E&Z modular-lab or Trasis AllinOne, to simply implement their modules with the component described in this paper to get an integrated solid target dissolution system. These components may also be compatible with commercial modules of other brands, making only small design/structural refinements (e.g., GE, IBA radiopharma solutions, ORA-NEPTIS, SCINTOMICS, IPHASE).

## Figures and Tables

**Table 1:** List of the dissolution system components.

Component	Material
Dissolution Vial	quartz; borosilicate glass; PEEK ^1^
Vial-Holder and mounting rods	aluminium alloy 6082; stainless steel
Target holder	aluminium; copper
O-ring	NBR ^2^ 70

^1^ PolyEther-Ether-Ketone. ^2^ Nitrile Butadien Rubber.

**Table 2:** Summary of the performed tests. Time refers to the total time which the process lasted.

Target	Pellet Mass/Thickness	Irradiation Parameters	Module	Vial Material	Process Parameters
^Nat^Y on Nb	0.0758 g 150 μm	12.5 MeV	Trasis	PEEK	2 mL HCl 2 M
		10 µA			RT ^1^
		5 min			Time 1 h
^Nat^Cr on Au/Cu	0.02 g 400 μm	16 MeV	E&Z	Borosilicate glass	3 mL HCl 8 M
		10 µA			Heating up to 70 °C
		15 min			Time 1 h
^Nat^Mo on Au/Cu	0.02 g 280 μm	19 MeV	E&Z	Borosilicate glass	1.5 mL (×3 times) H_2_O_2_ 30%
		1 µA			Heating up to 90 °C
		2 min			Time 30 min

^1^ Room temperature.

**Table 3:** Mean weight with standard deviation before and after dissolution of the tested targets. Accuracy interval of ±10^−4^ g.

Target	N Test	Target Weight		Difference (g)	Pellet’s Initial Weight (g)
		Before Dissolution (g)	After Dissolution (g)		
^Nat^Y on Nb	6	6.50 ± 0.04	6.43 ± 0.04	0.0758 ± 0.0007	0.0758
^Nat^Cr on Au/Cu	5	6.90 ± 0.02	6.70 ± 0.02	0.200 ± 0.002	0.2
^Nat^Mo on Au/Cu	3	6.86 ± 0.02	6.66 ± 0.02	0.200 ± 0.002	0.2

**Table 4:** Activity measured with dose calibrator at EOD and rescaled to EOB compared to the predicted activity at EOB calculated by using ISOTOPIA tool.

Isotope	Activity @ EOD (MBq)	Activity @ EOB (MBq)	
	Measured	Rescaled	Predicted
^89^Zr	8.8	8.9	9.7
^52^Mn	21	22	23
^99m^Tc	0.87	0.88	0.92

**Table 5:** MDA of the most prominent gamma lines for the contaminants ^197m/g^Hg and ^93m^Mo.

Backing	Pellet	Desired Isotope	Isotope Produced in the Backing	Half-Life (h)	E (keV)	MDA (Bq)
Nb	Y	^89^Zr	^93m^Mo	6.85	684.693	17.6
Cu/Au	Cr	^52^Mn	^197m^Hg	23.8	133.98	23.4
			^197g^Hg	64.14	191.364	1843.8
Cu/Au	Mo	^99m^Tc	^197m^Hg	23.8	133.98	10.4
			^197g^Hg	64.14	191.364	785.0

## Data Availability

Technical drawings of the reactor components are available from the corresponding author.

## Sample Availability

Samples are not available from the authors.

## References

[1] (2008). Cyclotron Produced Radionuclides: Principles and Practice.

[2] McCarthy D.W., Shefer R.E., Klinkowstein R.E., Bass L.A., Margeneau W.H., Cutler C.S., Anderson C.J., Welch M.J. (1997). Efficient Production of High Specific Activity 64Cu Using a Biomedical Cyclotron. Nucl. Med. Biol..

[3] Obata A., Kasamatsu S., McCarthy D.W., Welch M.J., Saji H., Yonekura Y., Fujibayashi Y. (2003). Production of Therapeutic Quantities of 64Cu Using a 12 MeV Cyclotron. Nucl. Med. Biol..

[4] Fonslet J., Tietze S., Jensen A.I., Graves S.A., Severin G.W. (2017). Optimized Procedures for Manganese-52: Production, Separation and Radiolabeling. Appl. Radiat. Isot..

[5] Pyles J.M., Massicano A.V., Appiah J.-P., Bartels J.L., Alford A., Lapi S.E. (2021). Production of 52Mn Using a Semi-Automated Module. Appl. Radiat. Isot..

[6] Kasbollah A., Eu P., Cowell S., Deb P. (2013). Review on Production of 89Zr in a Medical Cyclotron for PET Radiopharmaceuticals. J. Nucl. Med. Technol..

[7] Solbach C., Bertram J., Scheel W., Baur B., Machulla H., Reske S. (2013). Production of Zr-89 on a PETtrace Cyclotron 2013. J. Nucl. Med..

[8] Martini P., Boschi A., Cicoria G., Zagni F., Corazza A., Uccelli L., Pasquali M., Pupillo G., Marengo M., Loriggiola M. (2018). In-House Cyclotron Production of High-Purity Tc-99m and Tc-99m Radiopharmaceuticals. Appl. Radiat. Isot..

[9] Martini P., Boschi A., Cicoria G., Uccelli L., Pasquali M., Duatti A., Pupillo G., Marengo M., Loriggiola M., Esposito J. (2016). A Solvent-Extraction Module for Cyclotron Production of High-Purity Technetium-99m. Appl. Radiat. Isot..

[10] Bénard F., Buckley K.R., Ruth T.J., Zeisler S.K., Klug J., Hanemaayer V., Vuckovic M., Hou X., Celler A., Appiah J.-P. (2014). Implementation of Multi-Curie Production of 99mTc by Conventional Medical Cyclotrons. J. Nucl. Med..

[11] Synowiecki M.A., Perk L.R., Nijsen J.F.W. (2018). Production of Novel Diagnostic Radionuclides in Small Medical Cyclotrons. EJNMMI Radiopharm. Chem..

[12] Uccelli L., Martini P., Cittanti C., Carnevale A., Missiroli L., Giganti M., Bartolomei M., Boschi A. (2019). Therapeutic Radiometals: Worldwide Scientific Literature Trend Analysis (2008–2018). Molecules.

[13] Pupillo G., Mou L., Boschi A., Calzaferri S., Canton L., Cisternino S., De Dominicis L., Duatti A., Fontana A., Haddad F. (2019). Production of 47 Sc with Natural Vanadium Targets: Results of the PASTA Project. J. Radioanal. Nucl. Chem..

[14] Pupillo G., Mou L., Martini P., Pasquali M., Boschi A., Cicoria G., Duatti A., Haddad F., Esposito J. (2020). Production of 67Cu by Enriched 70Zn Targets: First Measurements of Formation Cross Sections of 67Cu, 64Cu, 67Ga, 66Ga, 69mZn and 65Zn in Interactions of 70Zn with Protons above 45 MeV. Radiochim. Acta.

[15] Boschi A., Martini P., Costa V., Pagnoni A., Uccelli L. (2019). Interdisciplinary Tasks in the Cyclotron Production of Radiometals for Medical Applications. The Case of 47Sc as Example. Molecules.

[16] Smilkov K., Janevik E., Guerrini R., Pasquali M., Boschi A., Uccelli L., Di Domenico G., Duatti A. (2014). Preparation and First Biological Evaluation of Novel Re-188/Tc-99m Peptide Conjugates with Substance-P. Appl. Radiat. Isot..

[17] Srivastava S.C. (2013). A Bridge Not Too Far: Personalized Medicine with the Use of Theragnostic Radiopharmaceuticals. J. Postgrad. Med. Educ. Res..

[18] Qaim S.M. (2019). Medical Radionuclide Production.

[19] Gagnon K., Wilson J.S., Holt C.M.B., Abrams D.N., McEwan A.J.B., Mitlin D., McQuarrie S.A. (2012). Cyclotron Production of 99mTc: Recycling of Enriched 100Mo Metal Targets. Appl. Radiat. Isot..

[20] Skliarova H., Cisternino S., Cicoria G., Marengo M., Palmieri V. (2019). Innovative Target for Production of Technetium-99m by Biomedical Cyclotron. Molecules.

[21] Skliarova H., Cisternino S., Cicoria G., Cazzola E., Gorgoni G., Marengo M., Esposito J. (2020). Cyclotron Solid Targets Preparation for Medical Radionuclides Production in the Framework of LARAMED Project. Journal of Physics: Conference Series.

[22] Pandey M.K., DeGrado T.R. (2020). Cyclotron Production of PET Radiometals in Liquid Targets: Aspects and Prospects. Curr. Radiopharm..

[23] Riga S., Cicoria G., Pancaldi D., Zagni F., Vichi S., Dassenno M., Mora L., Lodi F., Morigi M.P., Marengo M. (2018). Production of Ga-68 with a General Electric PETtrace Cyclotron by Liquid Target. Phys. Med..

[24] Oehlke E., Hoehr C., Hou X., Hanemaayer V., Zeisler S., Adam M.J., Ruth T.J., Celler A., Buckley K., Benard F. (2015). Production of Y-86 and Other Radiometals for Research Purposes Using a Solution Target System. Nucl. Med. Biol..

[25] Pandey M.K., Engelbrecht H.P., Byrne J.F., Packard A.B., DeGrado T.R. (2014). Production of 89Zr via the 89Y (p, n) 89Zr Reaction in Aqueous Solution: Effect of Solution Composition on in-Target Chemistry. Nucl. Med. Biol..

[26] Boschi A., Martini P., Pasquali M., Uccelli L. (2017). Recent Achievements in Tc-99m Radiopharmaceutical Direct Production by Medical Cyclotrons. Drug Dev. Ind. Pharm..

[27] IBA Pinctada Metal. https://www.iba-radiopharmasolutions.com/more-chemistry.

[28] ARTMS QIS. http://artms.ca/hardware-and-consumables.

[29] Comecer ALCEO. https://www.comecer.com/it/scopri-le-nuove-caratteristiche-alceo/.

[30] Gelbart W.Z., Johnson R.R. (2019). Solid Target System with In-Situ Target Dissolution. Instruments.

[31] Beaudoin J.-F., Tremblay S., Alnahwi A., Guerin B. (2019). Target Carrier and Dissolution System for Facilitating End-to-End in-Vault Cyclotron Production of Radiometals in Large Quantities. J. Nucl. Med..

[32] Esposito J., Bettoni D., Boschi A., Calderolla M., Cisternino S., Fiorentini G., Keppel G., Martini P., Maggiore M., Mou L. (2018). LARAMED: A Laboratory for Radioisotopes of Medical Interest. Molecules.

[33] Palmieri V., Skliarova H., Cisternino S., Marengo M., Cicoria G. (2018). Method for Obtaining a Solid Target for Radiopharmaceuticals Production. International Patent Application.

[34] Cicoria G., Pancaldi D., Lodi F., Malizia C., Costa S., Lucconi G., Lnfantino A., Zagni F., Fanti S., Boschi S. (2014). A Complete Compact Automatic Modular System for 64CuCl2 Production and Labelling of 64Cu-Tracers. European Journal of Nuclear Medicine and Molecular Imaging.

[35] Anselmi-Tamburini U. (2019). Spark Plasma Sintering. Reference Module in Materials Science and Materials Engineering.

[36] Hu Z.-Y., Zhang Z.-H., Cheng X.-W., Wang F.-C., Zhang Y.-F., Li S.-L. (2020). A Review of Multi-Physical Fields Induced Phenomena and Effects in Spark Plasma Sintering: Fundamentals and Applications. Mater. Des..

[37] Skliarova H., Cisternino S., Cicoria G., Marengo M., Cazzola E., Gorgoni G., Palmieri V. (2019). Medical Cyclotron Solid Target Preparation by Ultrathick Film Magnetron Sputtering Deposition. Instruments.

[38] IAEA ISOTOPIA. https://www-nds.iaea.org/relnsd/isotopia/isotopia.html.

